# Scaling of the GROMACS Molecular Dynamics Code to 65k CPU Cores on an HPC Cluster

**DOI:** 10.1002/jcc.70059

**Published:** 2025-02-13

**Authors:** Carsten Kutzner, Vedran Miletić, Karen Palacio Rodríguez, Markus Rampp, Gerhard Hummer, Bert L. de Groot, Helmut Grubmüller

**Affiliations:** ^1^ Theoretical and Computational Biophysics Max Planck Institute for Multidisciplinary Sciences Göttingen Germany; ^2^ Max Planck Computing and Data Facility Garching Germany; ^3^ Max Planck Institute of Biophysics Frankfurt am Main Germany

**Keywords:** benchmark, GROMACS, high performance computing, molecular dynamics, MPI

## Abstract

We benchmarked the performance of the GROMACS 2024 molecular dynamics (MD) code on a modern high‐performance computing (HPC) cluster with AMD CPUs on up to 65,536 CPU cores. We used five different MD systems, ranging in size from about 82,000 to 204 million atoms, and evaluated their performance using two different Message Passing Interface (MPI) libraries, Intel‐MPI and Open‐MPI. The largest system showed near‐perfect strong scaling up to 512 nodes or 65,536 cores, maintaining a parallel efficiency above 0.9 even at the highest level of parallelization. Energy efficiency for a given number of nodes was generally equal to or slightly better than parallel efficiency. We achieved peak performances of 687 ns/d for the 82k atom system, 116 ns/d for the 53M atom system, and about 35 ns/d for the largest 204M atom system. These results demonstrate that highly optimized software running on a state‐of‐the‐art HPC cluster provides sufficient computing power to simulate biomolecular systems at the mesoscale of viruses and organelles, and potentially small cells in the near future.

## Graphical abstract

We benchmarked the performance of the GROMACS 2024 molecular dynamics (MD) code on a modern high‐performance computing (HPC) cluster with AMD CPUs on up to 65,536 CPU cores with input systems ranging in size from 82,000 to 204 million atoms in size. The largest system showed near‐perfect scaling up to 65,536 cores, maintaining a parallel efficiency above 0.9 throughout.

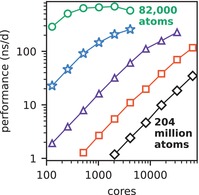

## 1 Introduction

Molecular dynamics (MD) simulations have become an essential and powerful tool for understanding, from fundamental physics, the molecular mechanisms of “biological nanomachines”, that is, proteins such as enzymes, ion channels, and ribosomes. While most protein simulations involve between 10,000 and several hundred thousand atoms, recent advances in computer hardware and software enable the study of increasingly larger systems [1, 2], with the current world record exceeding one billion atoms [3]. These developments suggest that it may soon be possible to simulate an entire biological cell, which would comprise 500 million to six billion particles [4]. Even for smaller systems, a significant challenge is adequately sampling the vast configuration space to make statistically robust statements about the system's average behavior. High‐Performance Computing (HPC) accelerates the collection of statistical data by (i) parallelizing individual simulations across multiple compute nodes [5] and (ii) running multiple similar copies of the system concurrently. While the latter is trivial, the former poses significant challenges in terms of algorithms and software engineering. To maximize individual simulation performance and scaling, MD packages such as NAMD [6], Amber [7], LAMMPS [8], GENESIS [3], CHARMM [9], OpenMM [10], or GROMACS [11] employ several levels of parallelization, including message passing (MPI) and shared memory (OpenMP) parallelization.

To distribute an MD system across many compute nodes, GROMACS divides the simulation volume into $N=n_{x}\times n_{y}\times n_{z}$ domains, with interactions between particles in a domain managed by a dedicated MPI rank. Each MPI rank can further utilize multiple OpenMP threads. In addition, separate MPI ranks [11] are used to compute the long‐range electrostatic interactions to mitigate the limited parallelizability of the Particle Mesh Ewald [12] (PME) method [13], which relies on all‐to‐all communication between the involved ranks. To overcome this limitation, Fast Multipole Methods (FMM) have been recently implemented instead [14, 15].

Splitting the simulation system into domains often leads to load imbalances, as the number or complexity of interactions is usually not the same between domains. A further load imbalance occurs when separate PME ranks are used. Automatic load balancing mechanisms and built‐in heuristics, such as estimating the optimal number of separate PME ranks, take much of the performance optimization burden off the scientist. However, when preparing for large‐scale simulations on expensive high‐end computing resources, it is generally advisable to compare the achieved performance with some kind of reference benchmark. Such reference performance numbers can also help to estimate the achievable simulation throughput and aid in the planning of new simulation campaigns.

Here we aim to provide such reference numbers for GROMACS for a large, modern, CPU‐based HPC platform, and at the same time to demonstrate the potential for simulating very large MD systems on state‐of‐the‐art hardware. We use the new AMD‐based supercomputer “Viper” [16] of the Max Planck Society, operated at the Max Planck Computing and Data Facility (MPCDF) in Garching.

## 2 Methods

In the last few years, AMD's EPYC CPUs have emerged as a competitor to Intel's Xeon family in the server and data center markets, including HPC. The competition between these two companies has driven innovation in the x86 CPU landscape, offering supercomputing centers more choices and driving advancements in performance, efficiency, openness, and cost‐effectiveness. The AMD‐based Viper HPC cluster has been operated by the MPCDF since July 2024. Each Viper node we used is equipped with two EPYC 9554 “Genoa” CPUs (128 physical cores, 3.1 GHz base frequency, turbo enabled) and 512 GB of RAM. The nodes are interconnected by an NVIDIA/Mellanox NDR200 (200 Gb/s) InfiniBand network with a non‐blocking fat‐tree topology. Further details on the hardware and software configuration can be found in the Viper user guide [16].

We used five different MD simulation systems for our benchmarks, ranging from 82,000 to 204 million atoms (Table 1). All of these are MD systems that have actually been or are being used in scientific projects and are not synthetic benchmarks. GROMACS .tpr input files for these systems are available at https://www.mpinat.mpg.de/grubmueller/bench. The MEM system has been used to study water permeation through an aquaporin tetramer that is embedded in a lipid bilayer [17]. The ribosome (RIB) system and similar ones are in current use for the study of the function of these protein factories in our cells [18, 19, 20, 21]. Systems such as PEP (“peptides”) have been used to study the oligomerization of steric zipper peptides, a process important for understanding the early stages of protein aggregation in many neurodegenerative diseases [19]. The largest systems are related to the human nuclear pore complex (hNPC) in the dilated conformation (PDB: 7R5J) [20]. The LUM system corresponds to the luminal ring of hNPC, while the SCF system is the hNPC scaffold consisting of the inner ring, nuclear ring, cytoplasmic ring, and luminal ring. Both systems are simulated in an explicit solvent with a salt concentration of 0.15 M NaCl. The NPC, arguably the largest protein assembly in human cells, consists of about 1000 proteins that form an 8‐fold symmetric pore in the nuclear envelope. Its main function is to regulate the exchange of molecules between the nucleus and the cytoplasm. The hNPC adjusts its diameter in response to changes in nuclear envelope tension, which affects transport [22, 23]. We are currently studying the hNPC scaffold and luminal ring dynamics to elucidate the molecular mechanism behind this structural flexibility and to gain key insights into its role in cellular regulation and disease.

**TABLE 1:** Specifications of the benchmark systems.

MD	# atoms	System size (nm)	Time‐step (fs)	Cutoff radii (nm)	PME grid spacing (nm)
MEM [17]	82k	10.8 × 10.2 × 9.6	2	1.0	0.12
RIB [18]	2M	31.2 × 31.2 × 31.2	4	1.0	0.135
PEP [19]	12.5M	50.0 × 50.0 × 50.0	2	1.2	0.16
LUM [20]	52.8M	166.3 × 144.0 × 21.8	2	1.1	0.16
SCF [20]	204.4M	166.4 × 144.1 × 84.2	2	1.1	0.16

For all benchmarks, we compiled GROMACS 2024.2 in mixed precision with AVX_512 SIMD instructions and OpenMP support, using the GCC 14.1.0 compiler. We built two versions, one with Intel‐MPI 2021.11 and a second one with Open‐MPI 4.1.6. Both MPI installations are based on UCX 1.16, which acts as a middle layer between the MPI library and the InfiniBand network hardware. For the fast Fourier transforms required by PME we use the FFTW library version 3.3.10 [24].

In our performance tests, we relied on the quite well‐performing empirical defaults for the parameters GROMACS can control, such as the number of separate PME nodes and whether or not to enable dynamic load balancing. We have generally refrained from extensive performance fine‐tuning, as this would not normally be done by practitioners. For systematic, in‐depth performance analysis and tuning, tools such as MDBenchmark [25] can help automate setup and analysis. One of the runtime settings that GROMACS cannot control is the partitioning of available CPU cores into MPI ranks and OpenMP threads, since the MPI processes are started outside of GROMACS. Since this can significantly affect performance, we benchmarked 1, 2, 4, and 8 OpenMP threads per MPI rank, always using all physical cores of a node, that is, in terms of ranks × threads per node 1 × 128, 2 × 64, 4 × 32, and 8 × 16. From these settings, we report the one that performed best. Simultaneous Multithreading (SMT) did not improve RIB performance on a single‐node (in fact, it was slightly worse with SMT), therefore we did not use SMT.

Each benchmark was run for 0.2–0.5 h of wall clock time. Performance was measured for the second half of the run because load balancing mechanisms take some time to reach optimal balance and because memory allocations tend to slow down the first few time steps.

For the Intel‐MPI benchmarks, we set the environment variable I_MPI_FABRICS=shm:ofi to explicitly enable shared memory transport (shm) for enhanced performance. Shared memory transport was temporarily disabled by default in Intel‐MPI, but will be enabled again in future releases starting with Intel‐MPI 2021.14.

To extract the power consumption of each GROMACS job, we used the “Smart Energy Manager Suite” (SEMS) provided by Eviden.

## 3 Results

**FIGURE 1:**
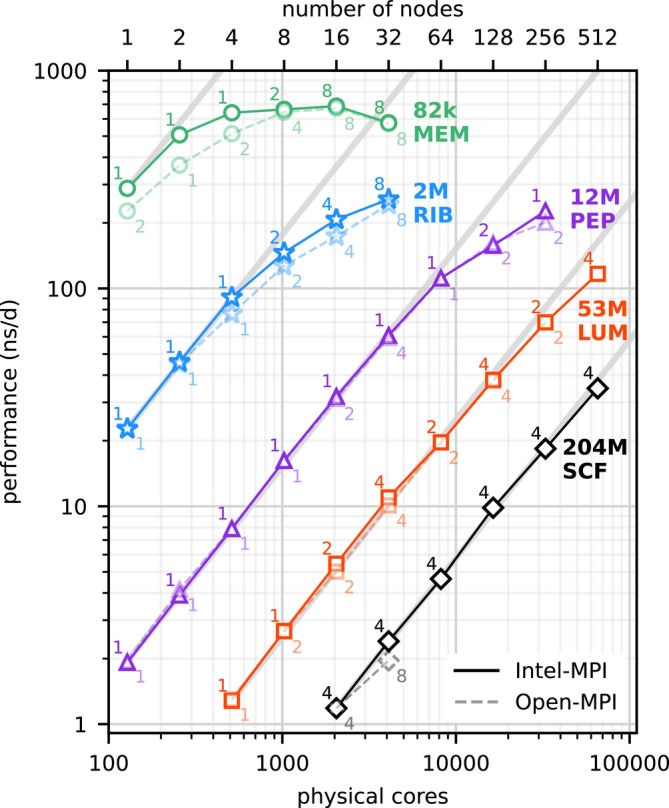
Strong scaling of GROMACS on up to 65,536 CPU cores. Measured performances of GROMACS 2024 on up to 512 Viper nodes with Intel‐MPI (solid) and Open‐MPI (dashed, semi‐transparent) for five MD systems (colors) ranging from 82k to 204M atoms in size. Numbers beside symbols refer to the optimal number of OpenMP threads per MPI rank. Diagonal gray lines indicate perfect scaling.

Figure 1 shows the strong‐scaling behavior for MD systems ranging from 82k to 204M particles between 128 and 65,536 cores (bottom axis) or one to 512 nodes (top axis), respectively. The largest 204M system closely follows the diagonal gray line, indicating near‐perfect scaling from four to 512 nodes. Even for the smaller systems (except MEM), scaling remains excellent for a smaller number of nodes. As expected, with decreasing system size, deviations from ideal scaling occur at lower node counts due to the comparatively smaller number of atoms per node and, hence also smaller workload per node.

We measured maximum performances of 687 ns/d for the 82k (MEM) system, 256 ns/d for the 2M (RIB) system, and 226 ns/d for the 12M (PEP) system on Viper, which, to our knowledge, are the highest performances ever reported for these specific benchmark systems, including on GPU‐accelerated nodes [11, 26, 27, 28, 29].

For systems larger than ten million atoms, the GROMACS performances for Intel‐MPI and Open‐MPI are very similar. For the smaller 82k and 2M systems, Intel‐MPI often outperforms Open‐MPI, with up to a 37% performance increase for the 82k system on two nodes, and up to a 19% increase for the 2M system on 16 nodes. For the 204M benchmark system, only Intel‐MPI results are available for ≥64 nodes, as all Open‐MPI runs were aborted due to memory limitations. If desired, benchmark results can probably be further improved for both MPI libraries by setting MPI runtime parameters optimized for the application and hardware setup. These runtime parameters would have to be determined beforehand by running MPI benchmarks.

The numbers next to the symbols in Figure 1 indicate the number of threads per MPI process that deliver the best performance. At low levels of parallelism, single‐threaded MPI ranks generally provide the best performance, avoiding any potential OpenMP overhead. However, as the total number of MPI ranks is increased in order to utilize more and more CPU cores, one will eventually reach a fundamental limit set by the cutoff radius. The latter limits the minimum domain size, which translates into a maximum number of domains (and hence MPI ranks) that can be used. When the maximum number of domains is reached, the only way to use more cores is to use more OpenMP threads for each MPI rank.

**FIGURE 2:**
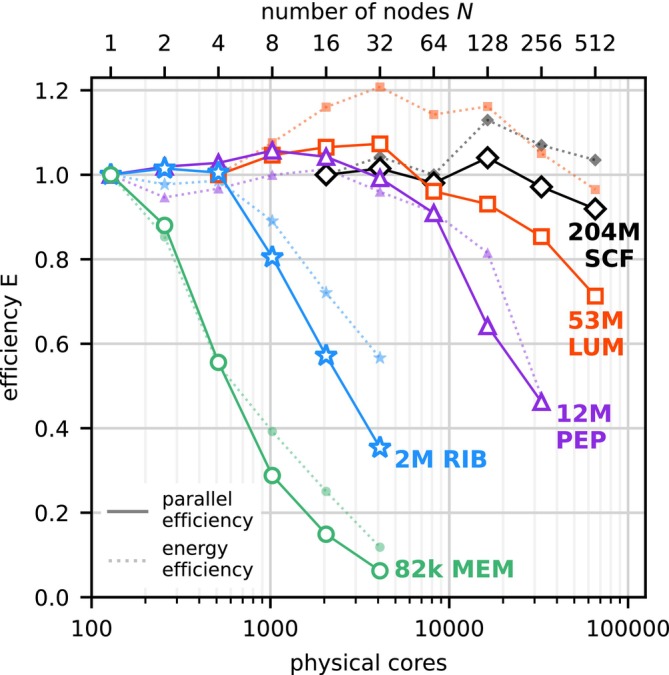
Parallel efficiency of GROMACS on up to 65,536 CPU cores. For the five benchmarked systems (colored), two efficiency metrics are shown for the Intel‐MPI benchmarks: (a) the parallel efficiency $E_{\text{par}}$ (Equation (1), solid lines), and (b) the normalized energy efficiency $E_{\text{en}}$ (Equation (2), dotted lines).

Figure 2 shows two metrics that indicate whether or not computer time is being used efficiently for a given number N of nodes. First, the parallel efficiency

(1) $$E_{\text{par}}(N)=\frac{P(N)}{N\cdot P(1)}$$

with P(N) denoting the performance on N nodes. Second, the energy efficiency, which is the performance on N nodes divided by the average power consumption pow(N) of the involved nodes,

(2) $$E_{\text{en}}(N)=\frac{P(N)}{\text{pow}(N)}$$

The plot shows the normalized energy efficiency, which is $E_{\text{en}}(N)$ divided by the energy efficiency at the smallest number of nodes that were tested.

For the small 82k system, the parallel efficiency $E_{\text{par}}$ drops relatively quickly, but for larger systems, $E_{\text{par}}$ remains above 0.8 even at very high levels of parallelization. If a parallel efficiency of at least 0.8 is targeted, the RIB system scales to about 2000 atoms per core, and the PEP system to 1500 atoms per core. For the PEP and LUM systems, we even observe a superlinear speedup ($E_{\text{par}}>1$) at medium node counts. This can occur especially in strong scaling scenarios, for example, when doubling the number of nodes causes computations to fit better into the CPU cache. At the highest parallelization of 512 nodes or 65,536 cores, the two largest systems still show decent ($E_{\text{par}}^{\text{LUM}}\approx 0.71$) to excellent ($E_{\text{par}}^{\text{SCF}}\approx 0.92$) efficiency, yielding remarkable simulation speeds of ≈116 ns/d and ≈35 ns/d, respectively.

Normalized energy efficiency generally follows the same trend as parallel efficiency. However, at higher node counts, energy efficiency often becomes higher than parallel efficiency. In the regimes where ideal scaling is observed in Figure 1, the power draw is about 1.2 kW per node^1^ which is constant over the runtime of the benchmark and varies only slightly between 1.1 kW per node for the larger and 1.3 kW per node for the smaller MD systems. Thus, in this regime, the total energy to the solution can simply be derived from the runtime and the number of compute nodes used. For example, the energy to solution for the large 204M system amounts to ≈(256·1.1kW)/(18.4ns/d)=367kWh/ns.

Where the simulation performance deviates from ideal scaling, the required power per node decreases as nodes become underutilized. For example, running the RIB system on 32 compute nodes consumes only about 1 kW per node. In the regime of ideal scaling on 1–4 nodes, RIB achieves an energy efficiency of 17.0–17.4 ns/d per kW. This was roughly the efficiency of GPU nodes five years ago and represents a threefold improvement over CPU nodes of that age [31].

## 4 Conclusions

Our GROMACS benchmarks on the new AMD‐CPU‐based HPC system of the Max Planck Society, Viper, for realistic and typical biomolecular systems show that the combination of powerful CPUs, a fast interconnect, and well‐tuned software makes it possible today to simulate extremely large MD systems with up to several hundred million atoms. For the largest system with 204 million atoms, the scaling behavior was shown to be close to ideal, with parallel efficiencies above 0.9 even at the highest parallelization levels (512 nodes with altogether 65,536 cores), while maintaining high energy efficiency. This demonstrates that modern HPC machines now provide the level of performance needed for simulating MD systems of the size of viruses, organelles, and eventually, small cells.

Besides the interconnect hardware, a high‐performance MPI communication library is crucial for enabling the highest GROMACS simulation performance at scale. While Intel‐MPI consistently delivered the best performance for our benchmarks, Open‐MPI is found to be a very competitive alternative.

## Data Availability

The data that support the findings of this study are available from the corresponding author upon reasonable request.

## Endnotes

^1^
For comparison, a single‐node High‐Performance‐Linpack run (AMD Zen HPL [30]) draws ca. 1.5 kW per node, which reflects GROMACS being a highly optimized, compute‐bound application.
